# Dendritic morphology of motor neurons and interneurons within the compact, semicompact, and loose formations of the rat nucleus ambiguus

**DOI:** 10.3389/fncel.2024.1409974

**Published:** 2024-06-12

**Authors:** Matthew J. Fogarty

**Affiliations:** Department of Physiology and Biomedical Engineering, Mayo Clinic, Rochester, MN, United States

**Keywords:** Golgi–Cox, brainstem, swallow, vocalisation, convex hull

## Abstract

**Introduction:**

Motor neurons (MNs) within the nucleus ambiguus innervate the skeletal muscles of the larynx, pharynx, and oesophagus. These muscles are activated during vocalisation and swallowing and must be coordinated with several respiratory and other behaviours. Despite many studies evaluating the projections and orientation of MNs within the nucleus ambiguus, there is no quantitative information regarding the dendritic arbours of MNs residing in the compact, and semicompact/loose formations of the nucleus ambiguus..

**Methods:**

In female and male Fischer 344 rats, we evaluated MN number using Nissl staining, and MN and non-MN dendritic morphology using Golgi–Cox impregnation Brightfield imaging of transverse Nissl sections (15 μm) were taken to stereologically assess the number of nucleus ambiguus MNs within the compact and semicompact/loose formations. Pseudo-confocal imaging of Golgi-impregnated neurons within the nucleus ambiguus (sectioned transversely at 180 μm) was traced in 3D to determine dendritic arbourisation.

**Results:**

We found a greater abundance of MNs within the compact than the semicompact/loose formations. Dendritic lengths, complexity, and convex hull surface areas were greatest in MNs of the semicompact/loose formation, with compact formation MNs being smaller. MNs from both regions were larger than non-MNs reconstructed within the nucleus ambiguus.

**Conclusion:**

Adding HBLS to the diet could be a potentially effective strategy to improve horses’ health.

## Introduction

The generation of aerodigestive behaviours such as swallowing and vocalisation must be coordinated with respiration and involve a highly orchestrated activation of skeletal muscle within the larynx, pharynx, and oesophagus, whose motor neurons (MNs) reside within the nucleus ambiguus (Lawn, 1966; Holstege et al., 1983; Davis and Nail, 1984; Bieger and Hopkins, 1987; Portillo and Pasaro, 1988a,b; Altschuler et al., 1991; McGovern and Mazzone, 2010). A somatotopic organisation of the rat nucleus ambiguus has been shown to prevail, with the more rostral compact formation innervating oesophageal MNs, with the more caudal semicompact and loose formations innervating the palatopharyngeal and laryngeal MNs, respectively (Fryscak et al., 1984; Bieger and Hopkins, 1987; Portillo and Pasaro, 1988b). Intense investigation of swallow and airway defence has identified a host of local brainstem patterns and premotor centres having projections to and from the nucleus ambiguus (Pitts et al., 2012; Bolser et al., 2015; Pitts and Iceman, 2023).

There are a variety of neural subtypes within the nucleus ambiguus, including cardiopulmonary neurons (McAllen and Spyer, 1978; Nosaka et al., 1979; Mazzone and Canning, 2013; Veerakumar et al., 2022) and extensive intermingling with the neurons of the ventral respiratory group (Ellenberger and Feldman, 1990a,b; Monnier et al., 2003; Neuhuber and Berthoud, 2022). Despite some strides being made in the molecular characterisation of various neurons within the nucleus ambiguus (Coverdell et al., 2022; Veerakumar et al., 2022), there is little quantified regarding the dendritic trees of the neuronal subtypes within the nucleus ambiguus. Indeed, extant data concern the general anatomical orientations of unspecified nucleus ambiguus MNs (Sun et al., 1995) or oesophageal MNs (Bieger and Hopkins, 1987; Altschuler et al., 1991; Kruszewska et al., 1994) and laryngeal MNs (Bieger and Hopkins, 1987), with no quantification of the dendritic or axonal processes, with all but one study in males alone and the outlier (Sun et al., 1995) having unspecified sexes.

In this study, we present the first detailed quantitative morphology (besides a limited Sholl analysis of seven neurons (Sun et al., 1995)) of MNs and interneurons within the rat nucleus ambiguus in male and female rats. We present these results stratified into anatomical locations within the rostral compact formation or more caudal semicompact and loose formations.

## Methods

### Ethical approval and experimental animals

All protocols were approved by the Mayo Clinic Institute Animal Care and Use Committee (IACUC #A57714) and complied with National Institutes of Health (NIH) and American Physiological Society guidelines. We used 10 female (five) and male (five) Fischer 344 rats of 6 months old obtained from Charles River. Rats were housed two rats per cage were housed under a 12 h: 12 h light–dark cycle with *ad libitum* access to food and water. The animals were allowed at least 1 week to acclimatise to these conditions before experiments were performed.

### Nissl terminal procedures, processing, imaging, and stereological counting

A subset (*n* = 6, 3 females, 3 males) of Fischer 344 rats were deeply anaesthetised with an intraperitoneal injection of ketamine (80 mg/kg) and xylazine (10 mg/kg) and intracardially perfused with saline (euthanised via exsanguination) followed by 4% paraformaldehyde in 0.1 M phosphate-buffered saline (PBS, pH 7.4). The fixed brainstem was then excised and post-fixed in 4% PFA in PBS overnight and then immersed overnight in 25% sucrose in PBS. Serial transverse 15 μm cryosections of the brainstem were cut and stained with 0.1% cresyl violet (v/v) in an acetic acid buffer using previously established methods (Fogarty, 2023). Brainstem images were created in a manner identical to previous studies (Fogarty, 2023) on a Zeiss Axioskop II equipped with a motorised stage 4x and 20x air objective (1.0 NA, Zeiss, Gottingen, Germany).

**Figure 1:**
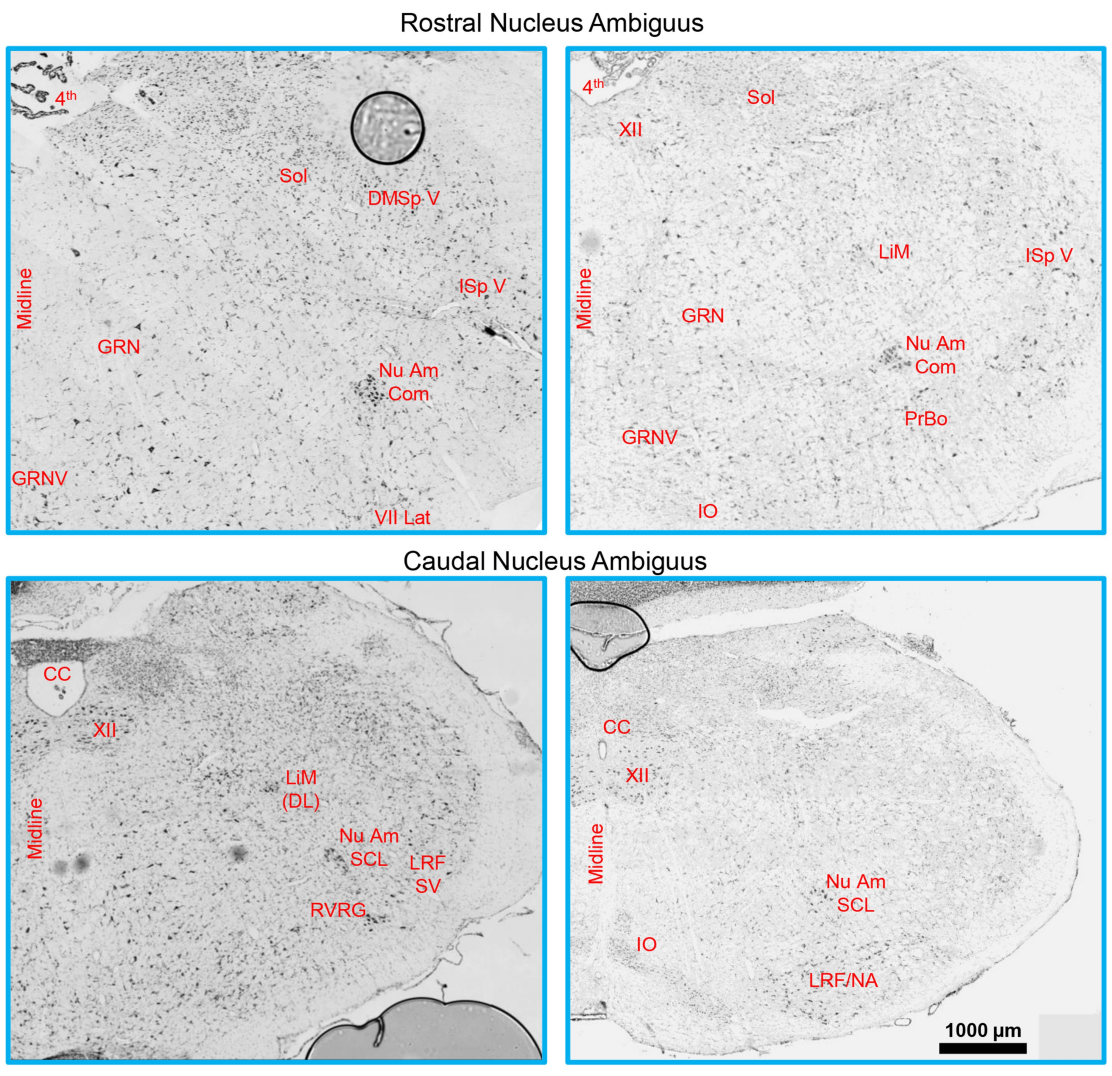
A Brainstem Nissl staining showing the rostral (top row) and caudal (bottom row) nucleus ambiguus identified. The compact formation of the nucleus ambiguus was relatively circular and started at the very caudal end of the lateral portion of the facial nucleus and extended to ~0.5–1 mm prior to the closure of the central canal. The semicompact/loose formations of the nucleus ambiguus were more ovoid in shape, commencing ~0.5–1 mm rostral to the formation of the central canal, and were immediately dorsal to the rostroventral respiratory group. 4th, fourth ventricle; CC, central canal; DMSp V, dorsomedial/spinal trigeminal nucleus; GRN, gigantocellular reticular nucleus; GRNV, ventral gigantocellular reticular nucleus; IO, inferior olive; ISp V, interpolar spinal trigeminal nucleus; LiM, linear nucleus of the medulla; LiM (DL), linear nucleus of the medulla (dorsolateral to main nucleus ambiguus); LRF/NA, lateral reticular formation noradrenaline cells; LRF SV, lateral reticular formation subtrigeminal region; Nu Am Com, compact formation of the nucleus ambiguus; Nu Am SCL, semicompact/loose formation of the nucleus ambiguus; PrBo, pre-pre-Bötzinger complex; RVRG, rostral ventral respiratory group; Sol, solitary tract; VII lat, lateral portion of the facial nucleus; XII, hypoglossal nucleus.

MN numbers were quantified unilaterally in the nucleus ambiguus, with landmark identification aided by a rat brain atlas (Paxinos, 1999; Paxinos and Watson, 2014) and past examples of retrogradely labelled rat laryngeal, pharyngeal, and oesophageal MNs (Wetzel et al., 1980; Hinrichsen and Ryan, 1981; Bieger and Hopkins, 1987; Portillo and Pasaro, 1988a; Altschuler et al., 1991; Flint et al., 1991; Sun et al., 1995; Hernandez-Morato et al., 2013). More specifically, the rostral boundary of the nucleus ambiguus comprised sections rostral to the hypoglossal nucleus, with the presence of the more rostral portion of the facial nucleus (Figure 1), consistent with reports from the cat (Holstege et al., 1983), and Sprague Dawley (Altschuler et al., 1991) and Wistar (Bieger and Hopkins, 1987; Paxinos and Watson, 2014) rats. The caudal extent of the nucleus ambiguus was approximated by that of the hypoglossal nucleus, within ~1 mm of the obex (consistent with the cat (Holstege et al., 1983; Holstege, 1989), and Sprague Dawley (Wetzel et al., 1980), Wistar (Hinrichsen and Ryan, 1981; Paxinos and Watson, 2014), and albino (Portillo and Pasaro, 1988a) rats) and complete enclosure of the central canal (Figure 1). As there was no readily identifiable demarcation between the rostral compact formation and the more caudal semicompact and loose formations, we approximated this border to be the halfway point between the most rostral extent of the nucleus ambiguus and the obex, consistent with reports in Sprague Dawley (Altschuler et al., 1991) and Wistar rats (Bieger and Hopkins, 1987; Paxinos and Watson, 2014). Occasionally (two of six rats used for Nissl), we would observe a cluster of larger neurons slightly medial (~10 degrees) and dorsal (~0.5–1 mm) from the nucleus ambiguus proper, usually in the section ~1 mm rostral to immediately after the obex in the longitudinal axis (Figure 1). These clusters are consistent with the dorsolateral group observed in rats (Wetzel et al., 1980; Hinrichsen and Ryan, 1981); however, we did not include these neurons in our quantifications as they do not project to laryngeal muscles (Hinrichsen and Ryan, 1981). We did not observe a dorsomedial cluster of neurons in these sections (Hinrichsen and Ryan, 1981).

Nucleus ambiguus MN counts were performed on every 10^th^ section in a manner identical to previous nucleus ambiguus histological studies (Sturrock, 1990) on a Zeiss Axioskop II equipped with a motorised stage 20x air objective (1.0 NA, Zeiss, Gottingen, Germany). To qualify for counting, MNs had to be large cells (the mean of the long and short axis > 15 μm) (Odutola, 1976; Dobbins and Feldman, 1995; Fogarty, 2023), have a dark cytoplasm, and have a distinct pale nucleus and dark nucleoli, as outlined previously (Lance-Jones, 1982; Fogarty et al., 2013, 2015). Note that despite our sectioning of Nissl material in an axis (transverse) non-parallel to the axis of the maximum somal projection of nucleus ambiguus, a prior study using retrograde approaches and transverse sectioning observed major diameters of MN somas of >30 μm (Hernandez-Morato et al., 2013), consistent with our >15 μm radii classification. Neuronal fragments, without a nucleus and nucleoli, are not counted in order to avoid “double counting,” consistent with stereological principles (Slomianka, 2020).

To estimate the MN surface area, the *x* and *y* radii of every hypoglossal MN counted were measured using ImageJ (Schneider et al., 2012), with surface areas calculated using the prolate spheroid approximation (Ulfhake and Cullheim, 1988).

### Golgi–Cox terminal procedures and processing

At the terminal experiment, a subset of animals (*n* = 4, 2 females, 2 males) were deeply anesthetised with an intraperitoneal injection of ketamine (80 mg/kg) and xylazine (10 mg/kg) and euthanised via exsanguination. Following euthanasia, the brainstem was removed and processed for Golgi–Cox impregnation (FD Rapid Golgi, FD NeuroTechnolgies) (Glaser and Van der Loos, 1981).

Following dissection, the brainstem was placed in Golgi–Cox impregnation solution for 16–18 days and changed once after 24 h. Following impregnation, the brainstem was frozen in melting isopentane and prepared for cryosectioning at 180 μm. Transverse-sectioned brainstem slices were left on slides to dry overnight at 24°C and then developed, dehydrated with ethanol and xylene, and coverslipped.

### Region selection, microscopy, and neuronal dendritic evaluation

The compact, semicompact, and loose regions of the nucleus ambiguus were located in transverse brainstem sections with the aid of aforementioned brain atlases and publications. Regions were also cross-referenced with brainstem histology from Fischer 344 rats in the present study (Figure 1). The nucleus ambiguus was imaged under brightfield illumination with a 40X oil objective (1.3 NA, 1600x 1,600 pixel array) using a pseudo-confocal (2 μm step size, 200 nm pinhole) method (Williams et al., 1994), allowing for sufficient illumination (non-fluorescent) of the Golgi-impregnated material. We used mosaic imaging to visualise multiple neurons within the region at high magnification, at the expense of some tessellating stitching artefacts. Despite Golgi–Cox assessments not being particularly amenable to stereological approaches, we endeavoured to adhere to the stereological principles, including systematic random sampling (Slomianka, 2020). Here, we ensured an equal sampling throughout the rostrocaudal axis of the compact, semicompact, and loose divisions. To aid consistency in the locations assessed, and the necessity for clustering of MNs to readily identify the nucleus ambiguus, interneuron dendritic assessments were only done in regions containing MNs.

Interneurons and MNs were assessed in a three-dimensional manner using Neurolucida 11 Desktop Edition (MBF Bioscience) in a manner identical to our past efforts using Golgi–Cox (Klenowski et al., 2016; Fogarty et al., 2016b, 2017b, 2019b, 2020b), with the dorsal or ventral and medial or lateral anatomical projections of the dendrites noted, similar to past studies in mice and rats (Kruszewska et al., 1994; Kanjhan et al., 2016). Neurons with multi-polar dendrites were classified as MNs only if they had a somal long radius of >15 μm, in accordance with prior histological studies and with retrograde approaches in rats (Bieger and Hopkins, 1987; Altschuler et al., 1991; Hayakawa et al., 1996; Fogarty et al., 2018; Williams et al., 2019; Fogarty, 2023). Thus, MNs and interneurons were distinguished based on somal size alone, not dendritic size. Dendrites were distinguished from axons via their tapering with branch order (Kruszewska et al., 1994; Kanjhan et al., 2016).

### Statistical methods

We used Prism 9 for all data analyses (GraphPad, Carlsbad, CA). Each data set was assessed for normality with D’Agostino and Pearson tests. *A priori* it was determined that within a particular data set, any data point outside 2.5 standard deviations from the mean was excluded from further analysis. Fortunately, we did not observe any outliers in the main outcome measures in the current study. Paired Student’s *t*-tests or unpaired or paired two-way ANOVA, with Bonferroni *post-hoc* tests, were performed, where appropriate. Statistical significance was established at the *p* < 0.05 level, with all *p*-values reported to four decimal places in the text. All data are reported as the mean ± 95% confidence intervals unless otherwise noted.

## Results

### Region selection and motor neuron quantification in transverse sections

**Figure 2:**
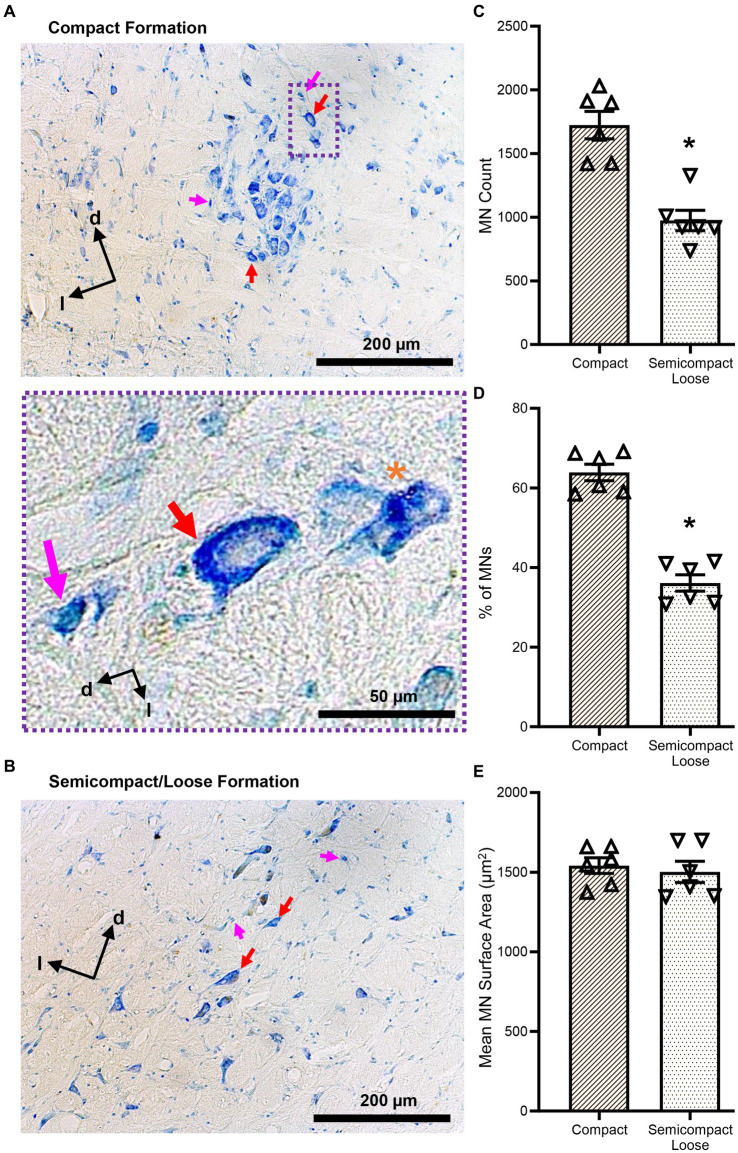
**(A,B)** Show brightfield images of Nissl-stained MNs within the compact and semicompact/loose formations, respectively, exhibiting classical histological characteristics of large cytoplasm and prominent nucleoli (red arrows). Non-MNs (putative interneurons), excluded from MN counts, are identified by pink arrows. Dorsal (d) and lateral (l) are also indicated on the images. The inset (purple dashed area) shows a higher powered image of an excluded non-MN (pink arrow) an example MN (red arrow), and a fragmented cell excluded from counting (orange asterisk). **(C)** Plots of reduced MN counts (mean ± 95% CI) in the semicompact/loose formations of the nucleus ambiguus compared to the compact formation. **(D)** Plots of reduced % of total MN (mean ± 95% CI) in the semicompact/loose formations of the nucleus ambiguus compared to the compact formation. **(E)** Plots of unchanged mean MN surface areas (mean ± 95% CI) between semicompact/loose formations and the compact formation of the nucleus ambiguus. All analyses were carried out using paired Student’s *t*-tests, *denotes statistical differences between groups (i.e., *p* < 0.05).

The compact, semicompact, and loose regions of the nucleus ambiguus were readily observed in female and male rats with the aid of a brainstem atlas (Paxinos, 1999; Paxinos and Watson, 2014) using Nissl staining (Figures 1, 2). These regions were matched to thicker Golgi–Cox-impregnated sections, where neurons meeting assessment criteria were traced and reconstructed in 3D.

The number of compact formation nucleus ambiguus MNs (1723 ± 277) exceeded that of semicompact/loose formation MNs (974 ± 205) by almost 2-fold (*p* = 0.0014, Student’s paired *t*-test; Figure 2C). Similarly, the percentage of total nucleus ambiguus MN within the compact formation (64 ± 5%) exceeded that of semicompact/loose formation MNs (36 ± 5%) by almost 2-fold (*p* = 0.0011, Student’s paired *t*-test; Figure 2D). There was no difference in the somal surface areas between nucleus ambiguus MNs form the compact formation (1,541 ± 127 μm^2^) compared to MNs for the semicompact/loose formations (1,502 ± 171 μm^2^, *p* = 0.6392, Student’s paired *t*-test; Figure 2E).

### Dendritic tree lengths in nucleus ambiguus motor neurons and interneurons

**Figure 3:**
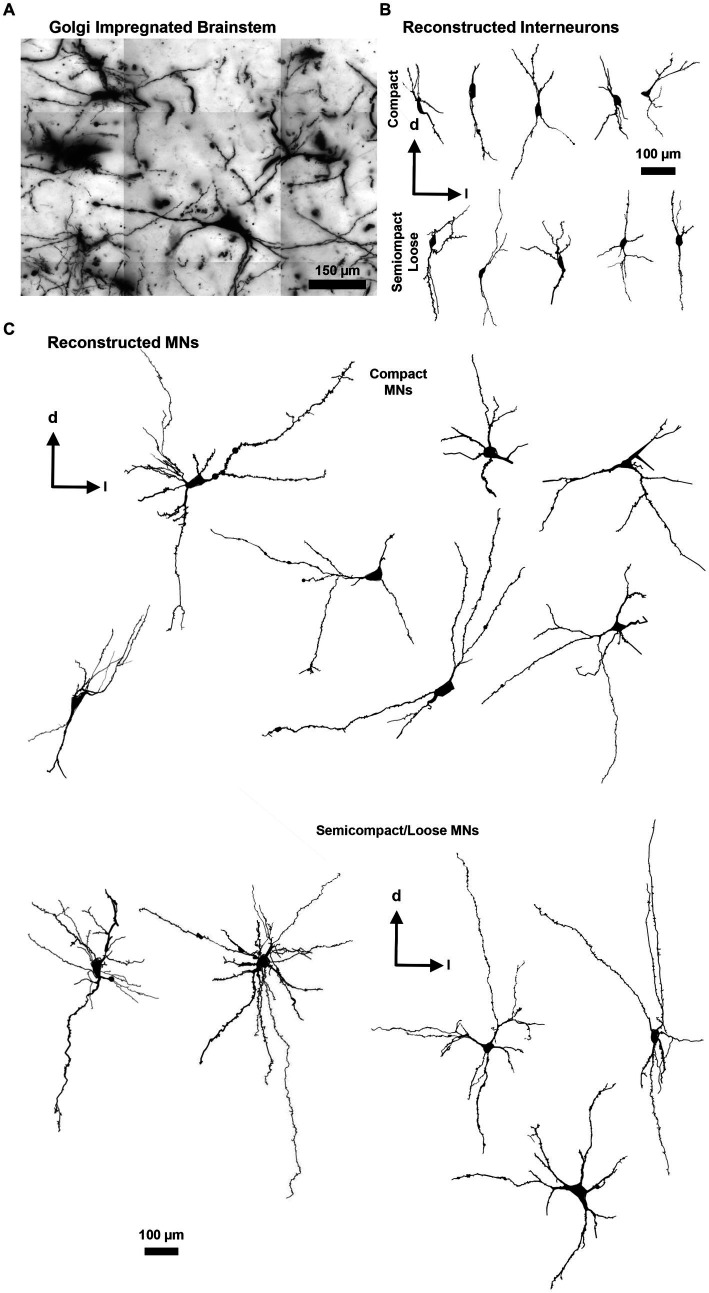
**(A)** Golgi-impregnated brainstem showing staining of nucleus ambiguus neurons. The tessellations are an artefact of the mosaic imaging process, this image is a minimum-intensity projection of five optical slices. **(B)** Representative Neurolucida tracings of nucleus ambiguus interneurons within the complex formation (top row) and the semicomplex/loose formations (bottom row). **(C)** Representative Neurolucida tracings of nucleus ambiguus MNs within the complex formation (top group) and the semicomplex/loose formations (bottom group). Dorsal (d) and lateral (l) are also indicated on the images.

**Figure 4:**
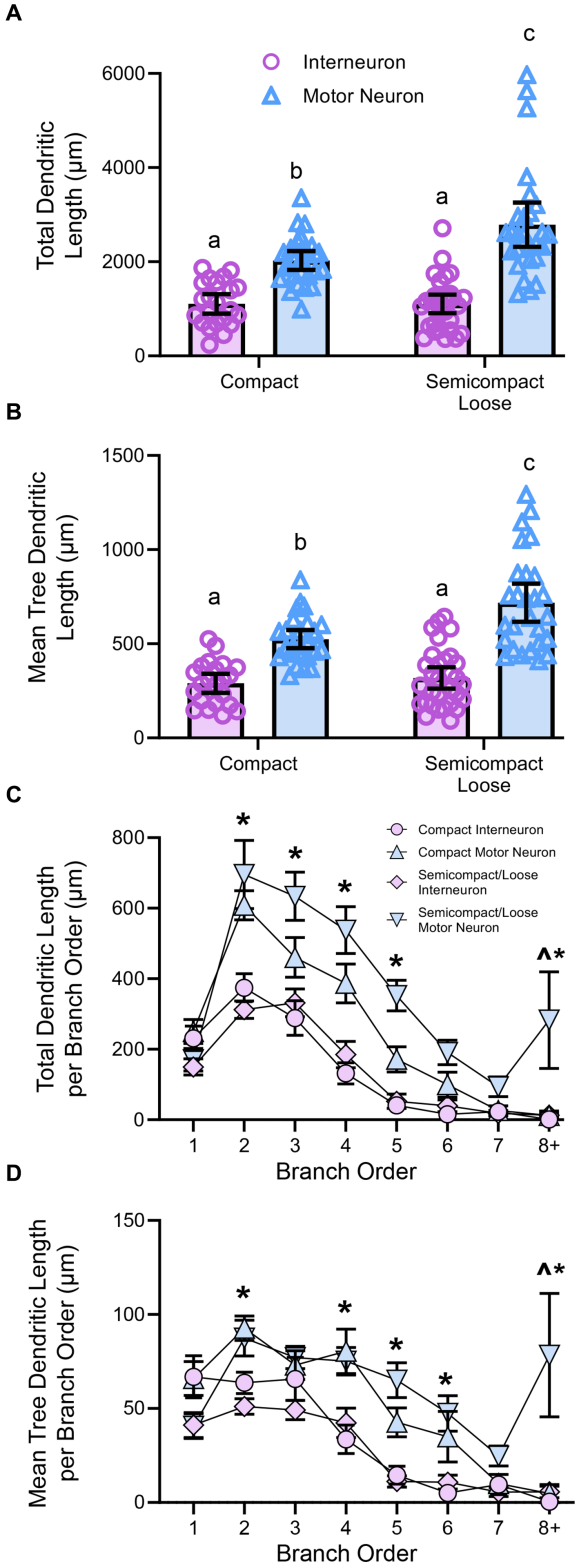
**(A)** Plots showing smaller total dendritic tree length (± 95% CI) in nucleus ambiguus interneurons compared to MNs, with semicompact/loose formation MN larger than compact formation MNs (two-way ANOVA with Bonferroni post-tests, with different letters denoting statistical differences between groups [i.e., *p* < 0.05]). **(B)** Plots showing smaller mean tree dendritic length (± 95% CI) in nucleus ambiguus interneurons compared to MNs, with semicompact/loose formation MN larger than compact formation MNs (two-way ANOVA with Bonferroni post-tests, with different letters denoting statistical differences between groups [i.e., *p* < 0.05]). **(C)** Plots of reduced total dendritic length per branch order of nucleus ambiguus interneurons compared to MNs; and reductions in compact MNs compared to semicompact/loose MNs at the eighth branch order and greater (three-way ANOVA with Bonferroni post-tests, *denotes the statistical difference between MNs and interneurons, ^denotes the statistical difference between compact MNs and semicompact/loose MNs [i.e., *p* < 0.05]). **(D)** Plots of reduced mean tree dendritic length per branch order of nucleus ambiguus interneurons compared to MNs; and reductions in compact MNs compared to semicompact/loose MNs at the eighth branch order and greater (three-way ANOVA with Bonferroni post-tests, *denotes statistical difference between MNs and interneurons, ^denotes statistical difference between compact MNs and semicompact/loose MNs [i.e., *p* < 0.05]). Dorsal (d) and lateral (l) are also indicated on the images.

Golgi–Cox impregnation readily labelled neurons within the nucleus ambiguus (Figure 3). Interneurons within the compact or semicompact/loose formations and MNs within the compact or semicompact/loose formations of the nucleus ambiguus were traced in 3D for morphometric quantifications (Figure 3). The total length of the dendritic arbour of nucleus ambiguus MNs and interneurons was dependent on the neuronal type (*F*_(1,103)_ = 81.6, *p* < 0.0001) and region (F_(1,103)_ = 6.9, *p* = 0.010, two-way ANOVA; Figure 4A). MNs within the compact formation (2027 ± 200 μm) had ~29% smaller total arbour lengths than MNs from the semicompact/loose formations (2,787 ± 468 μm; *p* = 0.0016, Bonferroni post-test; Figure 4A). Compact formation interneurons (1,107 ± 208 μm) exhibited ~half the total dendritic arbour length of compact (*p* = 0.0002) and semicompact/loose MNs (*p* < 0.0001, Bonferroni post-tests; Figure 4A). Similarly, semicompact/loose formations interneurons (1,107 ± 197 μm) exhibited ~half the total dendritic arbour length of compact (*p* = 0.0001) and semicompact MNs (*p* < 0.0001, Bonferroni post-tests; Figure 4A). There was no difference in total dendritic arbour length between interneurons of either region (*p* > 0.99, Bonferroni post-test; Figure 4A).

The mean length of each individual dendritic tree (tree length) of nucleus ambiguus MNs and interneurons was dependent on the neuronal type (*F*_(1,103)_ = 88.5, *p* < 0.0001) and region (F_(1,103)_ = 10.7, *p* = 0.0014; two-way ANOVA; Figure 4B). MNs within the compact formation (525 ± 47 μm) had ~25% smaller mean tree lengths than MNs from the semicompact/loose formations (718 ± 101 μm; *p* = 0.005, Bonferroni post-test; Figure 4B). Compact formation interneurons (290 ± 51 μm) exhibited ~45–60% the mean dendritic tree length of compact (*p* < 0.0001) and semicompact MNs (*p* < 0.0001, Bonferroni post-tests; Figure 4B). Similarly, semicompact/loose formations interneurons (318 ± 57 μm) exhibited ~50–65% the mean dendritic tree length of compact (*p* = 0.0001) and semicompact MNs (*p* < 0.0001, Bonferroni post-tests; Figure 4B). There was no difference in mean dendritic tree length between interneurons of either region (*p* > 0.99, Bonferroni post-test; Figure 4B).

When analysed with respect to neural branch order (from first to eighth and beyond), the total dendritic arbour length within a branch order was dependent on the branch order (*F*_(7,721)_ = 28.4, *p* < 0.0001), neuronal type (*F*_(1,103)_ = 42.9, *p* < 0.0001), and region*type interaction (F_(1,103)_ = 0.5, *p* = 0.4525; three-way ANOVA; Figure 4C). Bonferroni *post-hoc* tests show reduced dendritic length in nucleus ambiguus interneurons compared to MNs at second (*p* < 0.0001), third (*p* < 0.0001), fourth (*p* < 0.0001), fifth (*p* < 0.0001), and eighth and beyond (*p* = 0.0211) branch orders, regardless of region (Figure 4C). In addition, semicompact/loose formation MNs had more total dendritic arbour length at the eighth and beyond branch orders than compact formation MNs (*p* = 0.0130; Figure 4C).

When analysed with respect to neural branch order (from first to eighth and beyond), the mean dendritic tree length within a branch order was dependent on the branch order (F_(7,721)_ = 26.4, *p* < 0.0001), neuronal type (*F*_(1,103)_ = 42.9, *p* < 0.0001), and a region*type interaction (F_(1,103)_ = 4.3, *p* = 0.041; three-way ANOVA; Figure 4D). Bonferroni *post-hoc* tests show reduced mean tree dendritic length in nucleus ambiguus interneurons compared to MN at the at the 2^nd^ (*p* = 0.0025), 4^th^ (*p* = 0.0002), 5^th^ (*p* < 0.0001), 6^th^ (*p* = 0.0037), and 8^th^ and beyond (*p* = 0.0004) branch orders, regardless of region (Figure 4D). In addition, semicompact/loose formation MNs had more mean dendritic tree arbour length at the 8^th^ and beyond branch orders than compact formation MNs (*p* < 0.0001; Figure 4D).

### Dendritic tree surface areas in nucleus ambiguus motor neurons and interneurons

**Figure 5:**
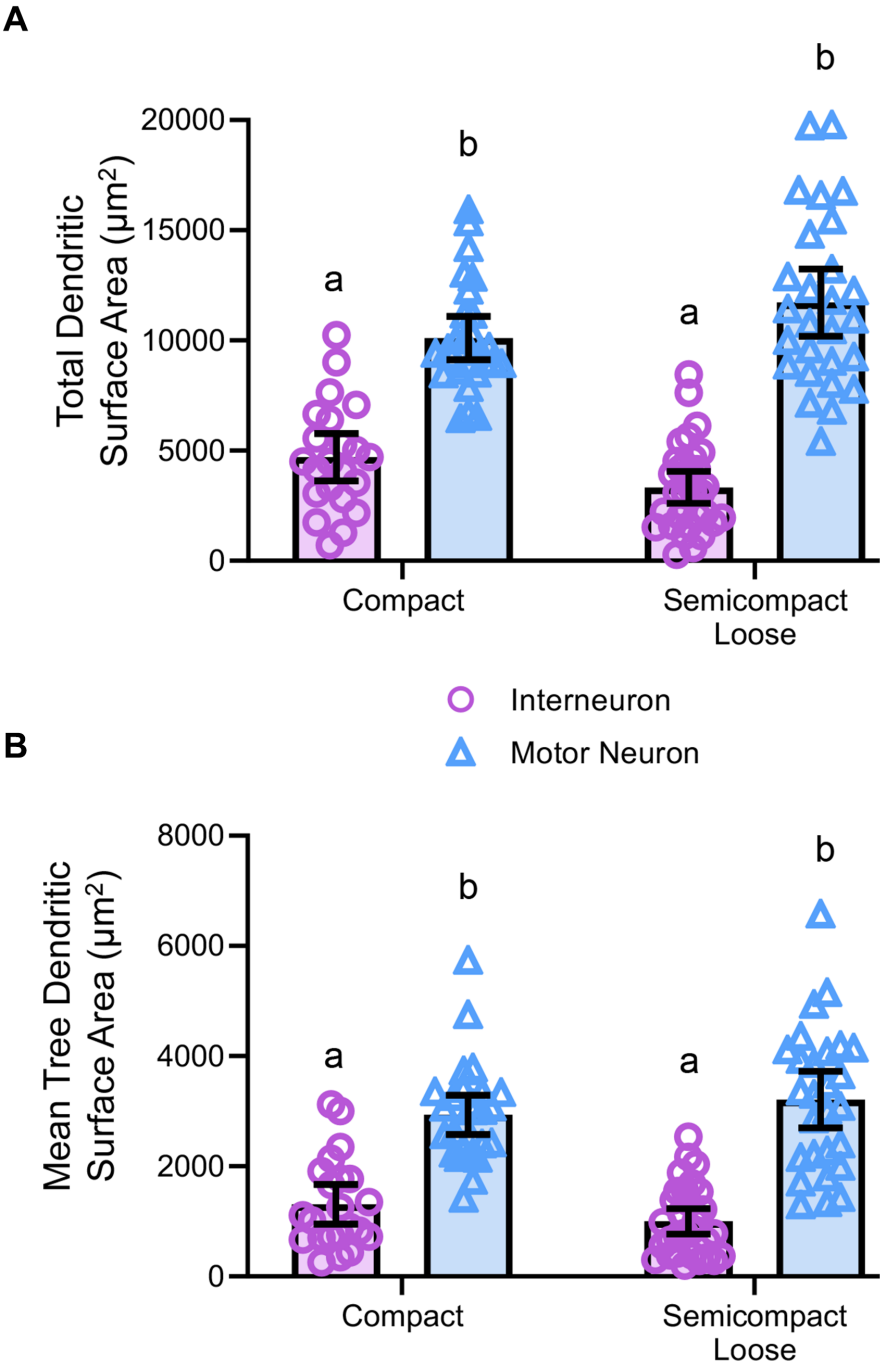
**(A)** Plots of smaller total dendritic surface area (± 95% CI) in nucleus ambiguus interneurons compared to MNs. **(B)** Plots of smaller mean tree surface areas (± 95% CI) in nucleus ambiguus interneurons compared to MNs. All analyses are two-way ANOVAs with Bonferroni post-tests, where appropriate. Different superscript letters denote statistical differences between groups (i.e., *p* < 0.05).

Dendritic diameters can be readily determined from Golgi–Cox-impregnated material (Figure 3A), with dendritic surface areas closely related to passive MN capacitance and intrinsic excitability (Rall, 1959, 1960, 1977; Ulrich et al., 1994). The total surface area of the dendritic arbour of nucleus ambiguus MNs and interneurons was dependent on the neuronal type (*F*_(1,103)_ = 163.9, *p* < 0.0001), but not region (F_(1,103)_ < 0.01, *p* = 0.8235, Figure 5A). MNs within the compact formation (10,111 ± 978 μm^2^) had greater surface areas than interneurons within the compact (4,700 ± 1,081 μm^2^; *p* < 0.0001) and semicompact/loose formations (3,330 ± 725 μm^2^, *p* < 0.0001, Bonferroni post-test; Figure 5A). Similarly, MNs within the semicompact/loose formations (11,733 ± 1,516 μm^2^) had greater surface areas than interneurons within the compact (*p* < 0.0001) and semicompact/loose formations (*p* < 0.0001, Bonferroni post-test; Figure 5A). There was no difference in the total dendritic surface area between MNs from different regions (*p* = 0.2085) nor between interneurons of different regions (*p* = 0.4717, Bonferroni post-test; Figure 5A). In particular, the range (semicompact/loose: 14369 μm^2^; compact: 9494 μm^2^) and interquartile range (semicompact/loose: 5938 μm^2^; compact: 2546 μm^2^) of the total dendritic surface area were markedly increased in semicompact/loose formation compared to compact formation MNs.

The mean dendritic tree surface area of nucleus ambiguus MNs and interneurons was dependent on the neuronal type (*F*_(1,103)_ = 109.9, *p* < 0.0001) but not region (F_(1,103)_ < 0.01, *p* = 0.9293, Figure 5B). MNs within the compact formation (2,936 ± 356 μm^2^) had greater mean tree surface areas than interneurons within the compact (1,309 ± 361 μm^2^; *p* < 0.0001) and semicompact/loose formations (1,001 ± 232 μm^2^; *p* < 0.0001, Bonferroni post-test; Figure 5B). Similarly, MNs within the semicompact/loose formations (3,212 ± 514 μm^2^) had greater mean tree surface areas than interneurons within the compact (*p* < 0.0001) and semicompact/loose formations (*p* < 0.0001, Bonferroni post-test; Figure 5B). There was no difference in the mean tree surface area between MNs from different regions (*p* > 0.99), nor between interneurons of different regions (*p* > 0.99, Bonferroni post-test; Figure 5B). In particular, the range (semicompact/loose: 14369 μm^2^; compact: 9494 μm^2^) and interquartile range (semicompact/loose: 1957 μm^2^; compact: 1111 μm^2^) of the mean tree dendritic surface area was markedly increased in semicompact/loose formation compared to compact formation MNs.

### Dendritic tree complexity in nucleus ambiguus motor neurons and interneurons

**Figure 6:**
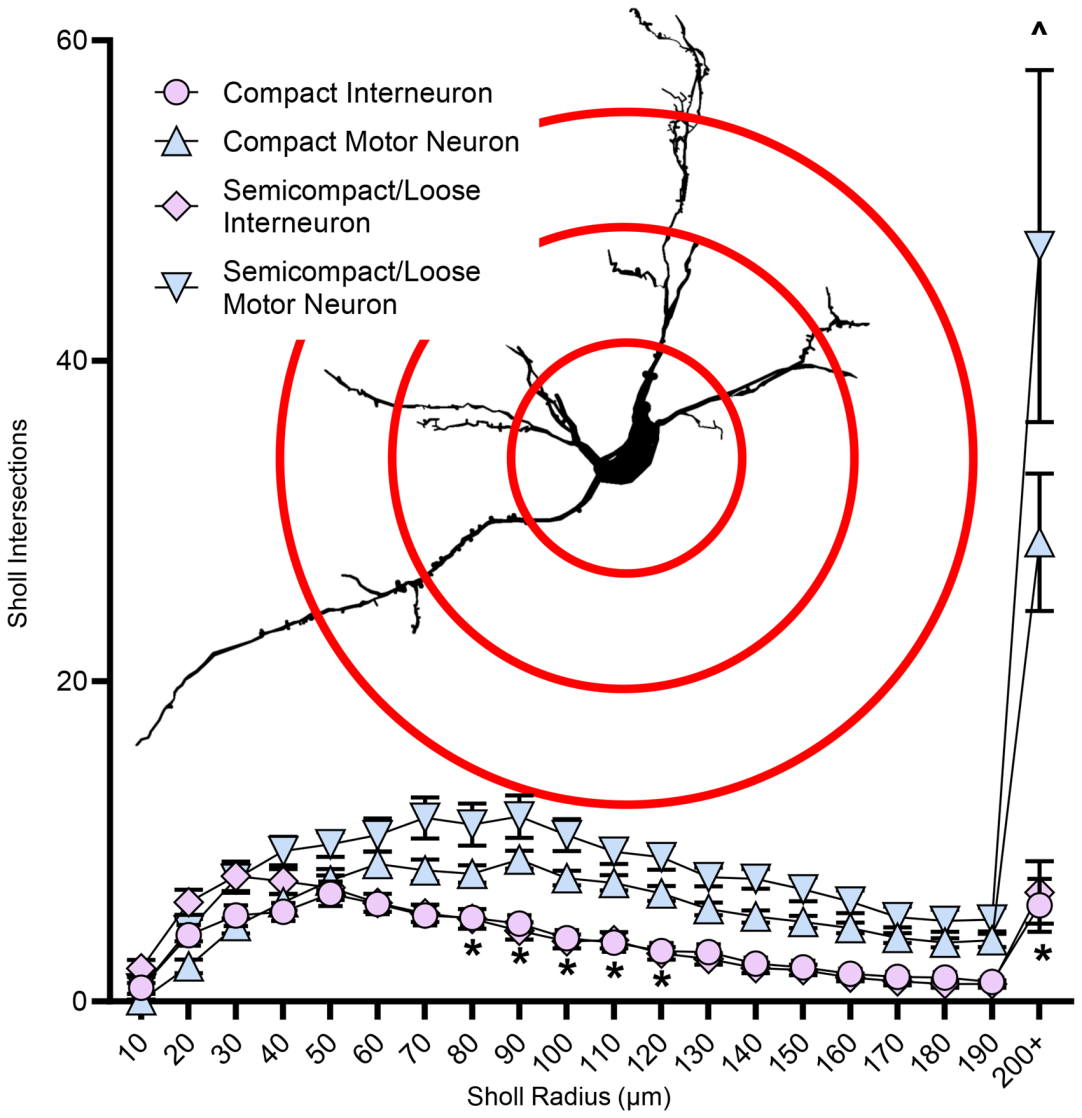
Plots of reduced dendritic Sholl interactions (mean ± SEM) in nucleus ambiguus interneurons compared to MNs, with semicompact/loose formation MNs having more Sholl interactions at very distal regions compared to compact formation MNs. Analysis three-way ANOVA with Bonferroni post-tests, *denotes statistical difference between MNs and interneurons, ^ denotes statistical difference between compact MNs and semicompact/loose MNs (i.e., *p* < 0.05). Insets show examples of Sholl analysis of a nucleus ambiguus MN; Sholl radii (red) are separated by 75 μm.

Sholl analysis of dendritic arbours provides an estimate of the complexity of a dendritic arbour (Sholl, 1953; Bird and Cuntz, 2019). We evaluated the number of intersections per Sholl radii at 10 μm intervals from the soma out to 200 μm and farther away from the soma (Figure 6). The number of intersections was dependent on the branch order (*F*_(19,1957)_ = 40.0, *p* < 0.0001), neuronal type (F_(1,103)_ = 54.3, *p* < 0.0001), and region*type interaction (F_(1,103)_ = 23.9, *p* = 0.0194; three-way ANOVA; Figure 6). Nucleus ambiguus MNs from the compact and semicompact/loose formations had greater interactions than compact formation interneurons and semicompact/loose formation interneurons from the 80^th^ to the 120^th^ μm, and the 200 μm and beyond radii (*p* < 0.04 in all cases, Bonferroni post-tests; Figure 6). Additionally, nucleus ambiguus MNs from the semicompact/loose formations had greater intersections than compact MNs at 200 μm and beyond radii (*p* < 0.0001, Bonferroni post-test; Figure 6).

### Dendritic tree convex hull assessments in nucleus ambiguus motor neurons and interneurons

**Figure 7:**
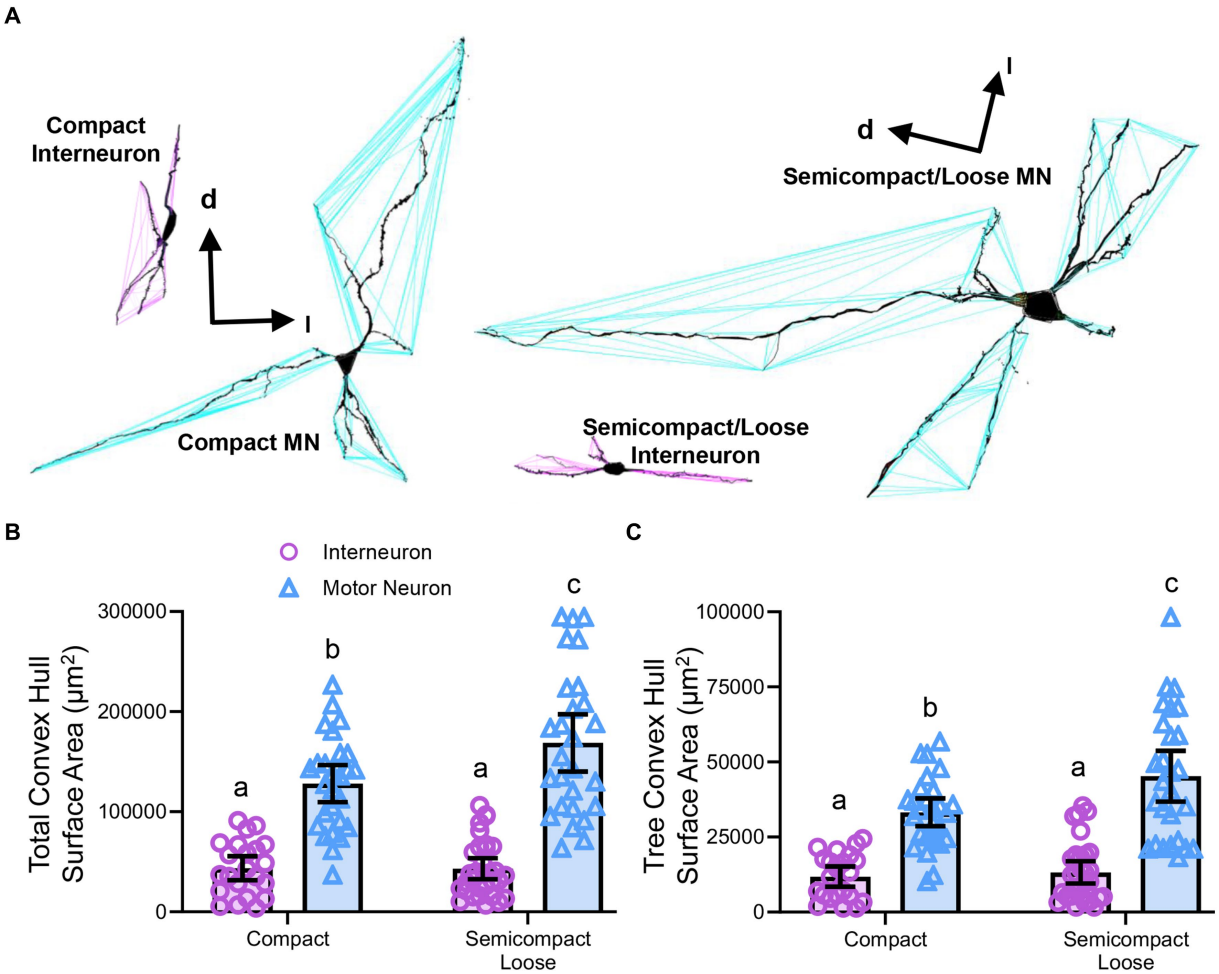
**(A)** 3D reconstruction of convex hulls (purple and blue polygons) of compact, semicompact/loose MNs and interneurons. **(B)** Plot shows smaller total dendritic tree convex hull surface areas (± 95% CI) in nucleus ambiguus interneurons compared to MNs, with semicompact/loose formation MN larger than compact formation MNs. **(C)** Plot shows smaller mean tree convex hull surface areas (± 95% CI) in nucleus ambiguus interneurons compared to MNs, with semicompact/loose formation MN larger than compact formation MNs. All analyses two-way ANOVAs with Bonferroni post-tests, with different letters denoting statistical differences between groups (i.e., *p* < 0.05). Dorsal (d) and lateral (l) are also indicated on the images.

Convex hull assessments are highly correlated with the overall field of inputs various neural types receive and thus an estimate of the receptive area (synapses and dendro–dendro contacts) of an individual neuron (Malmierca et al., 1995; Rojo et al., 2016; Bird and Cuntz, 2019) as opposed to an indication of passive computational properties (Marks and Burke, 2007). Convex hull structures were readily differentiated in nucleus ambiguus MNs and interneurons (Figure 7A). The total dendritic convex hull surface area of the dendritic arbours of nucleus ambiguus MNs and interneurons was dependent on the neuronal type (*F*_(1,103)_ = 128.0, *p* < 0.0001) and region (F_(1,103)_ = 4.7, *p* = 0.0332, Figure 7B). MNs within the compact formation (128,128 ± 18,564 μm^2^) had ~25% smaller total dendritic convex hull surface areas than MNs from the semicompact/loose formations (168,764 ± 28,687 μm^2^, *p* = 0.0135, Bonferroni post-test; Figure 7B). Compact formation interneurons (43,718 ± 12,017 μm^2^) exhibited ~24–35% of the total dendritic convex hull surface area of compact (*p* < 0.0001) and semicompact MNs (*p* < 0.0001, Bonferroni post-tests; Figure 7B). Similarly, semicompact/loose formations interneurons (43,158 ± 10,563 μm^2^) exhibited ~25–35% of the total dendritic convex hull surface area of compact (*p* < 0.0001) and semicompact MNs (*p* < 0.0001, Bonferroni post-tests; Figure 7B). There was no difference in the total dendritic convex hull surface area between interneurons of either region (*p* > 0.99, Bonferroni post-test; Figure 7B).

The mean dendritic tree convex hull surface area of each individual dendritic tree of nucleus ambiguus MNs and interneurons was dependent on the neuronal type (F_(1,103)_ = 98.1, *p* < 0.0001) and region (F_(1,103)_ = 6.2, *p* = 0.00144, Figure 7C). MNs within the compact formation (33,240 ± 4,638 μm^2^) had ~27% smaller mean dendritic tree convex hull surface area than MNs from the semicompact/loose formations (45,265 ± 8,479 μm^2^; *p* = 0.0114, Bonferroni post-test; Figure 7C). Compact formation interneurons (11,820 ± 3,398 μm^2^) exhibited ~25–35% of the mean dendritic tree convex hull surface area of compact (*p* < 0.0001) and semicompact MNs (*p* < 0.0001, Bonferroni post-tests; Figure 7C). Similarly, semicompact/loose formations interneurons (13,235 ± 3,689 μm_2_) exhibited ~30–40% the mean dendritic tree convex hull surface area of compact (*p* = 0.0001) and semicompact MNs (*p* < 0.0001, Bonferroni post-tests; Figure 7C). There was no difference in mean dendritic tree convex hull surface area between interneurons of either region (*p* > 0.99, Bonferroni post-test; Figure 7C).

## Discussion

Our study is the first to quantify the dendritic morphologies of MNs and interneurons within the compact, semicompact, and loose formations of the nucleus ambiguus. Our study had six major findings: (i) the number of MNs in the compact formation of the nucleus ambiguus exceeds those of the semicompact/loose regions; (ii) there were no systematic differences in MN size between different regions of the nucleus ambiguus; (iii) the dendritic lengths were greatest in MNs of the semicompact/loose regions than compact formation MN, with interneurons from both regions smaller than MNs; (iv) dendritic surface areas were greater in MNs than interneurons; (v) dendritic complexity was greatest in MNs of the semicompact/loose regions than compact formation MNs, with interneurons from both regions less complex than MNs; and (vi) dendritic convex hull area was greatest in MNs of the semicompact/loose regions than compact formation MNs, with interneurons from both regions less complex than MNs. These findings are interpreted in the context of the functional roles of the pharynx, larynx, and oesophagus during aerodigestive behaviours.

Delineation of the formations within the nucleus ambiguus is not straightforward, particularly in cases such as ours where retrograde labelling was not used; thus, we did not attempt to separate the semicompact from the loose formation. Nonetheless, given the inability of intramuscular approaches to quantify MN pools in their entirety (Mantilla et al., 2009), we are confident that our stereological approach provides a robust and reliable estimate of total nucleus ambiguus MNs (Sturrock, 1990; Slomianka, 2020) and a reasonable estimate of rostrocaudal regional differences. A major limitation of the present study is that our approach was not sensitive enough to observe MN somal size differences previously reported across regions (Hernandez-Morato et al., 2013). This is likely due to our underestimation of somal surface areas due to our sectioning being in the transverse, as opposed to the sagittal plane, where MN somal projections are greatest (Bieger and Hopkins, 1987). A reduced proportion of total nucleus ambiguus MNs within the semicompact/loose formation compared to the compact formation is consistent with prior reports (Sturrock, 1990; McGovern and Mazzone, 2010), and our total numbers resemble similar multiples (i.e., 2–2.5 times the MN number) of other brainstem nuclei in mice (Sturrock, 1990), previously observed in facial and hypoglossal MNs (Johnson and Duberley, 1998; Fogarty, 2023).

Overall surface areas of nucleus ambiguus MNs of Fischer 344 rats were slightly smaller than other brainstem (i.e., hypoglossal) and spinal cord (i.e., phrenic) MNs in Fischer 344 rats (Fogarty and Sieck, 2023). Although larger MNs are more likely to comprise fast fatiguable motor units compared to smaller MNs, comprising slow or fast fatiguable MNs (Burke et al., 1973; Dick et al., 1987; Heckman and Enoka, 2012; Fogarty and Sieck, 2019). This premise holds within rather than between motor pools, given the difference in MN sizes between different motor pools with muscles exhibiting mixed skeletal muscle fibre types (Brandenburg et al., 2018, 2020), particularly evident in brainstem MNs innervating orofacial muscles (Hinrichsen and Dulhunty, 1982; Rhee et al., 2004; Fogarty and Sieck, 2021; Sieck et al., 2023). Indeed, despite being smaller than hypoglossal MNs, laryngeal, pharyngeal, and oesophageal muscles express palpable levels of the myosin heavy chain type IIx (MyHC_2X_) (Rhee et al., 2004) or lower succinate dehydrogenase and mitochondrial volume density (Hinrichsen and Dulhunty, 1982), consistent with being fast intermediate (fatiguable) motor units (Brown et al., 2021b). Based on the past findings in aged Fischer 344 rats (Hashizume et al., 1988; Jacob, 1998; Kanda and Hashizume, 1998; Fogarty et al., 2018; Fogarty, 2023; Fogarty and Sieck, 2023) and facial (Johnson and Duberley, 1998) and findings in Fisher–Brown Norway crosses (Basken et al., 2012), we would expect nucleus ambiguus MNs to be vulnerable to age-associated death. This vulnerability would be consistent with age-associated dysphonias and dysphagias (Turley and Cohen, 2009; Basken et al., 2012; Marino and Johns, 2014; Thiyagalingam et al., 2021).

Our findings show that MNs within the nucleus ambiguus had much larger dendritic arbours than interneurons, regardless of whether they resided within the compact or semicompact/loose formations. This is not merely a function of somal size as interneurons in a variety of regions possess dendritic arbours that approach the lengths and projection of pyramidal neurons (Porter et al., 2001; Kecskes et al., 2013). These differences can be largely attributed to the more elaborate distal branches of brainstem MNs (Kruszewska et al., 1994; Sun et al., 1995; Hayakawa et al., 1996; Kanjhan et al., 2016; Fogarty et al., 2016a, 2017a,b, 2020b) compared to more simplistic interneurons (Hayakawa et al., 1996; Kubota et al., 2011; Klenowski et al., 2015). In addition, branch structure analyses showed an increased dendritic length of semicompact/loose MNs compared to compact formation MNs. These patterns of very large semicompact/loose formation MNs, large compact formation MNs and smaller interneurons were consistent with the dendritic complexity as evidenced by Sholl analysis. In addition to these passive properties, the receptive fields of neurons within the nucleus ambiguus held to an identical outline, with convex hull surface areas extremely large in semicompact/loose formation MNs, large in compact formation MNs, and smallest in the interneurons. Moreover, our quantitative data are consistent with qualitative data in transverse brainstem slices of adult Sprague Dawley rats labelled with neurobiotin (Sun et al., 1995), where dendritic restrictions within the compact formation MNs were noted, compared to the more elaborate projections of laryngeal MNs within the semicompact formation.

In the present study, we did not examine the activities of the individual neurons, nor did we examine circuit activation and motor or cardiorespiratory responses. However, an abundance of prior retrograde tracing studies provides us with a reasonable idea of the normal physiological role of each neural population. These studies provide some clues as to why the morphologies of the MNs differ across regions within the nucleus ambiguus. As aforementioned, the MNs within the compact formation innervate the oesophagus (Holstege et al., 1983; Fryscak et al., 1984; Bieger and Hopkins, 1987; Altschuler et al., 1991), whilst the more caudal nucleus ambiguus MNs of the semicompact and loose formations innervate the palate and larynx (Hinrichsen and Ryan, 1981; Holstege et al., 1983; Flint et al., 1991; Hayakawa et al., 1996; Basken et al., 2012). Despite eschewing retrograde labelling, being largely incompatible with our Golgi approach, we used these extensive prior characterisations, the first-rate transverse-orientation rat brain atlas (Paxinos and Watson, 2014), and our cross-matching of histology in the Fischer 344 rat (this study). We therefore suggest that anatomical variability does not affect our study to a greater extent than the widely appreciated intermingling of neurons (particularly the ventrolateral respiratory group) within the semicompact/loose formations previously identified (Ellenberger and Feldman, 1990b; Sun et al., 1995). Although the nucleus ambiguus MN dendrites are reported to project in a longitudinal (parasagittal) manner (Bieger and Hopkins, 1987; Altschuler et al., 1991; Kruszewska et al., 1994; Hayakawa et al., 1996), two of these papers illustrate large transverse projections (Bieger and Hopkins, 1987; Altschuler et al., 1991), and one paper shows non-compact formation neuron, consistent with a semicompact/loose MN having extensive dorso-ventral projections (consistent with being oriented in the transverse plane) (Kruszewska et al., 1994). In addition, a prior study using intracellular labelling showed extensive arbourisation of nucleus ambiguus MNs in the transverse plane (Sun et al., 1995). Thus, given the advantages of anatomic orientation provided by transverse sectioning (Figure 1), we proceeded with this approach—although our neuronal arbourisation quantifications may be an underestimate, particularly of total arbours as some trees of the compact formation MNs may have been obscured in the z-axis. Our final major limitation is the shrinkage of brainstem sections during processing (Kruszewska et al., 1994), which may hinder the classification of MNs, although this likely puts MNs in the interneuron category, due to reductions in size below the 30-μm-diameter MN threshold (Bieger and Hopkins, 1987; Altschuler et al., 1991; Hayakawa et al., 1996; Fogarty et al., 2018; Fogarty, 2023). Thus, our interneuron groups within the nucleus ambiguus dendritic assessments may include some of the smaller MNs. Indeed, these interneurons may be parasympathetic neurons, visceral and branchial efferents, gamma MNs, or shrunken small alpha MNs (Kalia and Mesulam, 1980; Hinrichsen and Ryan, 1981; Stuesse and Fish, 1984; Portillo and Pasaro, 1988a). Despite gamma MNs being important in other MN pools, it is unlikely that the smaller neurons in the population within the nucleus ambiguus contain gamma MNs as proprioception and muscle spindles are not readily identified in rat laryngeal muscles (Andrew, 1954, 1955, 1956a). So more correctly, the interneurons may be considered putative interneurons or non-MNs.

In the MNs of the compact formation, there is an overall need to coordinate oesophageal functions with ventilation. The close proximity of the compact region MNs and the relative restriction of their length and convex hull surface areas compared to semicompact/loose MNs is consistent with the necessity for synchronous activation of the cervical oesophagus and coordinated propagation in the thoracic and abdominal regions of the oesophagus (Altschuler et al., 1991; Kruszewska et al., 1994). The lack of extensive dendritic lengths at eighth and beyond branch orders is also consistent with this premise. This oesophageal activation in swallow is likely highly stereotyped in action [i.e., a reflex binary, initiated synchronously by distension or by the initiation of the swallow pattern (Andrew, 1956b; Meyer and Castell, 1982; Paterson, 1999; Broussard and Altschuler, 2000)], similar to the laryngeal functions in ventilation and swallow (Andrew, 1955; Fregosi and Ludlow, 2014; Huff et al., 2022; Pitts and Iceman, 2023). By contrast, laryngeal muscles operate over a wider range of behaviours (notably changes in pitch during vocalisation) (Wetzel et al., 1980; Bartlett and Knuth, 1984; Jurgens, 2009; Riede et al., 2020) and emotional states (Geyer et al., 1978; Geyer and Barfield, 1978; McIntosh et al., 1978; Barfield et al., 1979; Vanderhorst et al., 2000; Holstege, 2014), particularly in humans (Levin, 2006; Pisanski et al., 2022). Synchronous activation of MNs and their innervated muscle can be facilitated via: (i) gap junctions, which are not prevalent in oesophageal nucleus ambiguus MNs (Kruszewska et al., 1994); (ii) homogeneous high intrinsic excitability (i.e., less dendritic length/surface area leading to reduced neural capacitance), evident in findings of reduced dendritic surface areas and narrower ranges dendritic surface area in compact, compared to semicompact/loose MNs; and (iii) constrained dendritic projections, integrating inputs from fewer brainstem areas, evident in the reduced Sholl and dendritic convex hull surface areas of compact, compared to semicompact/loose MNs.

What remains evolutionarily peculiar is the observation that these oesophageal-innervating MNs are rostral to both pharyngeal MNs and the disparate brainstem swallow centres within the nucleus of the solitary tract and the dorsal and ventral swallow groups (Holstege et al., 1983; Kessler and Jean, 1985a,b; Zheng et al., 1997; Jean, 2001; Huff et al., 2022; Pitts and Iceman, 2023). Second, these neurons are activated last in the swallow phase—first oral, then pharyngeal, then oesophageal (Pitts and Iceman, 2023). Oesophageal activity seems impervious to unilateral dysfunction, due to a bilateral spread of innervated fibres within the oesophagus (Gruber, 1978). Moreover, in these rats, biochemical analysis of fibres is consistent with type IIa fibres, relatively resilient to age-associated weakness and denervation (Fogarty et al., 2019a, 2020a; Brown et al., 2021a; Sieck et al., 2023). Thus, the oesophageal functions and phase of swallow may be less vulnerable to perturbations of age and degeneration than those involved in the pharyngeal and oral phases. In particular, the resilience of compact formation nucleus ambiguus is evident in aged mice, where ~20% death of compact formation retrofacial nucleus ambiguus MNs compared to an overall loss of ~35% (Sturrock, 1990). In brainstem and spinal MNs of rats and humans, there are known motor unit type-dependent differences in vulnerability, with slow and fatigue-resistant MNs resilient and fatigueable MNs prone to death in old age (Hashizume et al., 1988; Kanda and Hashizume, 1998; Fogarty et al., 2018; Fogarty, 2023) and neurodegenerative conditions (Kiernan and Hudson, 1991; Pun et al., 2006; Dukkipati et al., 2018). Type-dependent differences in dendritic arbours, with larger MNs having larger arbours MNs (Cullheim et al., 1987; Ma and Vacca-Galloway, 1991; Leroy et al., 2014; Fogarty et al., 2019b, 2020b) and synaptic inputs, larger MNs having more excitatory inputs (Kellerth et al., 1979; Brannstrom, 1993) exist in MNs and may underpin differential susceptibility to degeneration and death (Ahmed et al., 2016; Fogarty, 2018; Kiernan et al., 2019). Our current observation of relatively restricted dendritic arbour size and previous quantifications of less synaptic inputs compared to pharyngeal MNs (Hayakawa et al., 1996) is consistent with a propensity of this region to harbour MNs comprising fatigue-resistant (slow or fast) motor units. It remains to be determined whether compact formation MNs and their dendritic arbours are conserved with age and neurodegeneration in the rat. We would expect that in old age, compact formation MNs would be spared in number and degenerative phenotype.

The MNs of the semicompact/loose formations, innervating the palate and laryngeal muscles, are activated during vocalisation and the oral and pharyngeal phases of swallow (Wetzel et al., 1980; Holstege et al., 1983; Holstege, 1989; Vanderhorst et al., 2000; Jurgens, 2009). Indeed, interactions between emotional processing centres of the brainstem and the muscles of speech/vocalisation are becoming increasingly studied (Vanderhorst et al., 2000; Holstege, 2014; Subramanian et al., 2018, 2021). Similarly to oesophageal MNs, these MNs must coordinate their activation with the control of breathing as well as vomiting, cough, and a host of other behaviours (Kessler and Jean, 1985b; Car and Amri, 1987; Tomomune and Takata, 1988; Amri et al., 1991; Nishino and Hiraga, 1991; Dick et al., 1993; Umezaki et al., 1998; Jean, 2001; Sawczuk and Mosier, 2001; Roda et al., 2002; Pitts et al., 2012; Fregosi and Ludlow, 2014; Huff et al., 2022). Thus, compared to oesophageal MNs, palate and laryngeal MNs must integrate their activities across a wider range of behavioural states. The exaggerated length and convex hull surface area to provide the substrate for synaptic inputs from diverse sources is evident in the current study and is consistent with synaptic inputs quantified by electron microscopy and qualitative assessments in Sprague Dawley rats (Sun et al., 1995; Hayakawa et al., 1996). The larger range of dendritic sizes is consistent with a more diverse motor unit population, confirmed by the mixed myosin heavy chain expressions confirming type I, IIa, IIx, IIb, and mixtures of IIx and IIb with extraocular fibres in cricothyroid, cricoarytenoid, and thyroarytenoid muscles (Rhee et al., 2004). Thus, within the semicompact/loose formations of the nucleus ambiguus there resides age- and neurodegeneration-vulnerable and resilient MNs innervating the larynx, similar to other mixed motor unit population motor pools (Fogarty et al., 2018; Fogarty, 2023; Fogarty and Sieck, 2023). This is borne out in observations of dysphagia and dysphonia in the elderly, in Alzheimer’s disease and amyotrophic lateral sclerosis sufferers (Turley and Cohen, 2009; Humbert et al., 2010; Christmas and Rogus-Pulia, 2019; Garand et al., 2022), alongside rodent studies of aged Fischer 344–Brown Norway crosses (Basken et al., 2012; Krekeler et al., 2020). In particular, the study by Basken showed a specific loss of retrogradely labelled laryngeal MNs (Basken et al., 2012), although it remains unconfirmed whether these findings hold in Fischer 344 rats. It is likely that vulnerable MNs in this population will exhibit distal dendritic pathology consistent with degeneration.

## Conclusion

In conclusion, this study presents the first comprehensive quantitative evaluation of the dendritic morphologies of MNs and non-MNs within the compact, semicompact, and loose formations of the nucleus ambiguus. Our findings in MNs are consistent with motor unit type-specific differences between the different regions. Our findings are also consistent with differences in the number of different behaviours each motor pool must coordinate its activities with. These findings provide a solid basis with which to assess various developmental, ageing, and disease states.

## Data availability statement

The original contributions presented in the study are included in the article/supplementary material, further inquiries can be directed to the corresponding author.

## Ethics statement

The animal study was approved by Mayo Clinic Institute Animal Care and Use Committee (IACUC approval #A57714). The study was conducted in accordance with the local legislation and institutional requirements.

## Author contributions

MF: Conceptualization, Data curation, Formal analysis, Funding acquisition, Investigation, Methodology, Project administration, Resources, Software, Supervision, Validation, Visualization, Writing – original draft, Writing – review & editing.
